# Resveratrol Promotes Foot Ulcer Size Reduction in Type 2 Diabetes Patients

**DOI:** 10.1155/2014/816307

**Published:** 2014-02-20

**Authors:** Yuriy K. Bashmakov, Samir H. Assaad-Khalil, Myriam Abou Seif, Ruzan Udumyan, Magdy Megallaa, Kamel H. Rohoma, Mohamed Zeitoun, Ivan M. Petyaev

**Affiliations:** ^1^Lycotec Ltd, Granta Park, Platinum Building, Cambridge CB21 6GP, UK; ^2^Department of Internal Medicine, Faculty of Medicine, Alexandria University, Alexandria 21527, Egypt; ^3^Department of Clinical Pathology, Faculty of Medicine, Alexandria University, Alexandria 21527, Egypt; ^4^Clinical Epidemiology and Biostatistics, Örebro University Hospital and School of Health and Medical Sciences, Örebro University, Örebro, Sweden

## Abstract

*Objective*. The effect of a proprietary formulation of *trans*-resveratrol (*t*-RSV) on manifestations of diabetic foot syndrome (DFS) was studied in type 2 diabetic patients with newly diagnosed diabetic foot ulcers. *Method*. Placebo-controlled, examiner-blinded, parallel-group randomized controlled pilot clinical trial (ACTRN Clinical Trial Registry number 12610000629033) involving 24 patients with DFS (15 males and 9 females, average age of 56.4 ± 9.1 years) divided into the placebo and RSV-treatment groups was performed. 50 mg of *t*-RSV or placebo capsules was given to each patient twice a day over a 60-day time period. *Results*. Reduction in the parameters reflecting diabetic ulcer size was more profound in the RSV group as compared to placebo. RSV-treated patients also had a marginally improved performance in the foot pressure test. A statistically significant decline in the plasma fibrinogen level, but not CRP, was also found in the RSV-treated patients. Some improvement in the plasma lipid profile and fasting glucose levels were not related to RSV-treatment, since they have been seen on both the RSV and placebo groups, revealing the effectiveness of medical supervision and education in the newly diagnosed patients with DFS. *Conclusion*. *t*-RSV supplementation promotes reduction of the foot ulcer size and reduces plasma fibrinogen level in type 2 diabetic patients.

## 1. Introduction 

The International Diabetes Federation estimates that there are currently 285 million diabetic patients worldwide. This represents a twofold increase in the number of individuals affected by diabetes since 2000 [1]. A further increase in the number of diabetic patients of ~7 million per year is projected for the near future. Up to 25% of diabetics are likely to develop diabetic foot syndrome at some time during the course of their disease [2, 3]. In general terms, diabetic ulcers represent the most severe and persistent cause of chronic ulceration in the human body. Poorly managed diabetic foot syndrome eventually leads to lower limb amputation [4]. Overall life expectancy of patients with diabetic foot syndrome, even in industrialized countries, is reduced by at least 10 years [5]. Five-year mortality rates in newly diagnosed patients might reach 55% and rise to 74% after lower limb amputation [6, 7]. These remarkable values exceed known mortality rates for breast, colon, and prostate cancers [8].

There are significant deficiencies in the understanding of the pathophysiological mechanisms behind diabetic ulceration. It is generally believed that neuropathy, peripheral arterial disease, and infection are the three major determinants of diabetic foot syndrome [9]. Although a large array of biochemical factors are suspected to be involved in abnormal wound healing in diabetes, there are no known specific molecular or biochemical markers for diabetic foot ulcers to distinguish them from other types of ulcer [10].

The association between plasma concentration of acute-phase response mediators (IL-6, CRP, and fibrinogen) and severity of diabetic foot syndrome has been reported to be of the most significance [11].

Wound closure is a primary goal in diabetic foot syndrome treatment. Foot pressure relief, wound debridement, and infection control are the most widely accepted options in the treatment of diabetic foot patients [12]. To date, a pharmacological approach to wound healing has been almost nonexistent. Current treatment modalities are limited to topical application of growth factors and/or bioengineered substitutes for human skin [13]. A multidisciplinary approach and new pharmacological options for diabetic ulcer treatment are urgently needed [14].

In the current paper, we report the effect of *trans*-resveratrol (*t*-RSV), a polyphenolic compound, on the healing rate of foot ulcers in type 2 diabetes patients. A proprietary formulation [15] of *t*-RSV (*t*-RSV-Lycosome) was used in this study. The working hypothesis and study rationale were thoroughly discussed in our previous paper [16].

## 2. Materials and Methods

### 2.1. Patients

A trial comparing the effect of resveratrol against a placebo group was set up. The study initially included 31 patients. Seven of them dropped out of the study for different reasons not related to the general goal and specific aims of the study protocol. Twenty-four patients who had documented history of type 2 diabetes with stable glycemic control during the previous 3 months of disease and newly diagnosed diabetic ulcers lasting for at least 4 weeks were randomized into a resveratrol-treatment group and a control (placebo) group according to age and gender. Patients were followed up at the outpatient clinic of the Diabetes Foot Center established at Alexandria University (Egypt) by the International Diabetes Federation. In the treatment group (*n* = 14), there were 8 men and 6 women, aged from 34 to 74 years with an average of 54.0 ± 10.1 years and diabetes duration averaging 15.0 ± 6.9 years. In the control group (*n* = 10), there were 7 men and 3 women aged from 47 to 67 years with an average of 59.8 ± 6.6 years and diabetes duration averaging 15.2 ± 9.5 years. The standardized protocol was approved by the Alexandria Faculty of Medicine Ethical Committee. Each patient had been informed as to the purpose of the study and had signed a consent form. The trial was conducted in accordance with the Helsinki Declaration and registered at ACTRN Clinical Trial Registry (number 12610000629033).

### 2.2. Study Outcome and Objectives

The main outcome of the study was to determine if 60-day treatment with a proprietary oral formulation of *trans*-resveratrol (Lycotec Ltd, Cambridge, UK) led to the reduction of foot ulcer size in type 2 diabetic patients. Objectives of the study included evaluation of performance of the patients in a foot pressure test and assessment of fasting plasma glucose level as well as proinflammatory markers (CRP and fibrinogen) in blood before and after treatment with resveratrol in comparison to the placebo group.

### 2.3. Inclusion Criteria

The study initially included patients with newly diagnosed diabetic ulcers lasting at least 4 weeks (sized from 0.5 to 2 cm, graded 1 or 2 on the Wagner scale) and having documented history of type 2 diabetes. All patients were willing and able to maintain previously established treatment for diabetes and a similar hygiene regimen of body and foot care throughout the trial period.

### 2.4. Exclusion Criteria

Diseases which affect the development and outcomes of diabetic foot syndrome (cancer, AIDS/HIV, chronic trauma, nutritional abnormalities, etc.); heart failure and stroke, cardiovascular and nephrological diseases requiring immediate medical intervention; infectious complications of diabetic foot syndrome (osteomyelitis, abscess, and phlegmona); critical abnormalities of blood circulation in the limb (ischemia or venous insufficiency, thrombophlebitis); distal/proximal necrosis of the limb tissues; pregnancy and/or breastfeeding.

Participants were scheduled for follow-up visits every 2 weeks for intermediate evaluation and ulcer debridement. Detailed physical examination and laboratory assessment were performed at the baseline and end point of the study (the 60th day of treatment).

### 2.5. Treatment Protocol

All patients underwent a similar treatment protocol based on the International Consensus on the Diabetic Foot [17] which included offloading, ulcer debridement, and infection control. One capsule of proprietary formulation of resveratrol containing 50 mg of active substance (*t*-RSV-L, Lycotec Ltd, UK) was given to each patient twice a day (morning and evening) after a meal with noncarbonated water over a 60-day time period. Control patients were assigned to take a placebo pill which contained only inert excipients used in the resveratrol pill formulation.

### 2.6. Patient Evaluation

Initial medical examination included detailed anamnestic, clinical, and laboratory evaluation. Marital and educational status medical history including individual history of diabetes mellitus as well as type of treatment received was established. Examination of the foot was scrupulously performed. Vascular assessment, ulcer size and depth, presence of infection, and sensation were investigated in each patient.

Vascular evaluation was done by registering of pedal pulses. Ulcer size was measured by multiplying the largest by the second largest perpendicular diameter of the skin lesion. Cumulative ulcer size (sum of the two largest perpendicular diameters and ulcer depth) was also computed for each individual patient. Depth of the ulcer was defined as either superficial or deep. Infection was diagnosed if edema, erythema, regional lymph node enlargement, pain, or fever was present. Neuropathy presence was evaluated by pressure sensation test, blunt/sharp discrimination, vibration sensation, and tactile test. Patients were considered positive for infection or neuropathy if at least two of the above listed features were present. According to their location, ulcers were defined as plantar or nonplantar. In regard to duration, ulcers were determined as acute (up to 2 weeks), subacute (from 2 weeks to 8 weeks), and persisting (over 2 months). Moreover, each foot assessed was graded according to the Wagner classification [18].

Among diabetes-related diseases and disorders traced in the patients during the trial were hypertension (diagnosed when systolic/diastolic blood pressure was ≥140/90 mmHg or by the use of any antihypertensive medication), retinopathy (documented by ophthalmological evaluation), nephropathy (defined as macroalbuminuria or renal failure), and hyperlipidemia (acknowledged when total cholesterol was ≥200 mg/dL, LDL cholesterol was ≥120 mg/dL, and triglycerides were ≥150 mg/dL or by use of any lipid lowering medication). Finally, each patient was orally instructed on diabetic foot care and given a brochure designed by the Alexandria Diabetic Foot Center.

### 2.7. Laboratory Measurements

Routine hematology and urine analysis were performed on each patient. Fasting blood samples were collected from the patients by venipuncture on the day following admission to the outpatient clinic and on the day after completion of the 60-day treatment period. EDTA plasma was separated and the specimens were stored at −80°C prior to analysis. Plasma levels of glucose, lipids (total cholesterol, HDL-cholesterol, LDL-cholesterol, and triglycerides), CRP, and fibrinogen were determined using standard biochemical methods. HOMA-IR values were calculated using HOMA-IR calculator (version 0.3).

### 2.8. Foot Pressure Test

Peak plantar pressures were recorded during level barefoot walking using the MatScan system (Tekscan, Boston, MA, USA). This system consists of a 5 mm thick floor mat (432 mm × 368 mm) incorporating 2,288 resistive sensors (1.4 sensors/cm^2^) sampling at a rate of 40 Hz. The two-step gait initiation protocol was used to obtain foot pressure data, as it requires fewer trials than the mid-gait protocol and has similar retest reliability [19]. Three trials were recorded, which is sufficient to ensure adequate reliability of pressure data [20]. Research Foot software (version 5.24) was used to determine peak pressures for the whole foot as well as under the forefoot, the midfoot, and the heel [21].

### 2.9. Statistical Analysis

Between-group differences at one time point were evaluated by Wilcoxon-Mann-Whitney test (continuous variables) and Fisher's exact test (categorical variables). Paired *t*-test was used to evaluate the within-group mean differences over the study period. For CRP, median differences were calculated. Multiple linear regression was used to see if the change in ulcer size as well as foot plantar pressure after 60 days of trial was different between treatment groups.

Data analysis was performed using Stata SE, version 12.1. All statistical tests were two-sided and statistical significance level Alpha was set at 0.05 for all analysis.

## 3. Results

### 3.1. Baseline Data

**Figure 1:**
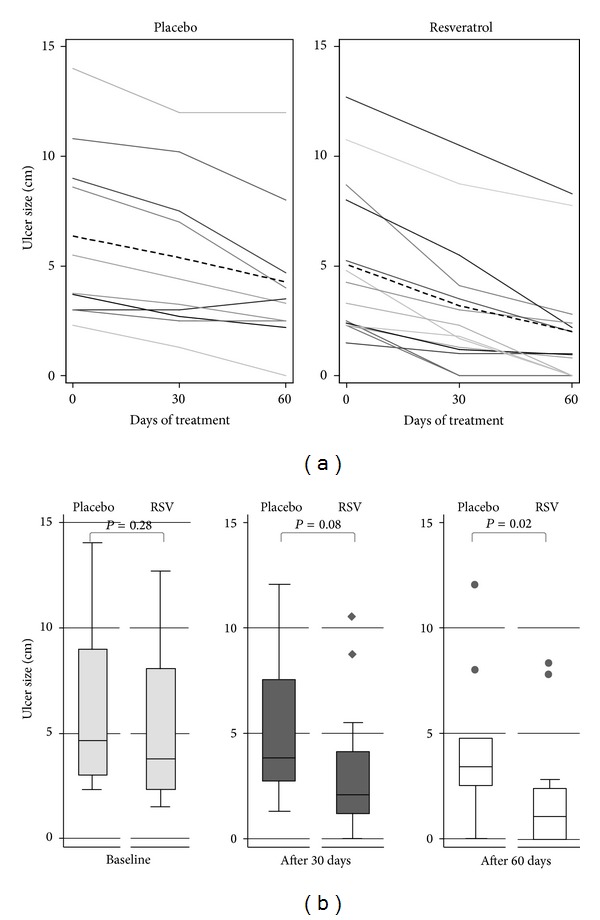
(a) Cumulative ulcer size of 10 patients in placebo and 14 patients in resveratrol groups over study period. Plots show ulcer size of each patient (solid lines) and mean ulcer size (interrupted bold line) in the study period over 60 days. (b) Ulcer size at baseline and after 30 and 60 days of treatment (*P* values from Wilcoxon-Mann-Whitney tests).

Table 1 summarizes the general characteristics of the patients enrolled in the trial. All patients had type 2 diabetes and two-thirds of them were males. The groups were similar with respect to cumulative ulcer size at baseline (*P* = 0.30) with median IQR values of 3.8 (2.3–8.0) cm in the treatment and 4.6 (3–9) cm in the control group (Figure 1). Neuropathy was present, whereas the infection was absent in all patients. The similarities in ulcer size and frequency of diabetes-related complications as well as duration of foot ulceration between the two major groups suggest an acceptable level of randomization.

### 3.2. Changes in Ulcer Size

The major observation made in this trial was related to changes in parameters reflecting the foot ulcer size. All patients in the RSV group and 90% in the placebo group enrolled in the study had some positive dynamics upon completion of the 60-day trial. However, the groups differed in both the dynamics and magnitude of the changes. Surface area of diabetic foot ulcers in the placebo group dropped from pre-treatment median of 4.25 cm^2^ (95% CI: 1.5, 16.00) to 2.75 cm^2^ on 30th day (95% CI: 1.00, 12.59) and 2.13 cm^2^ (95% CI: 0.66, 7.38) at the end point of the study. Reduction in the ulcer surface area was more significant in the RSV group. Ulcer surface area fell in the RSV-treated patients from 2.63 cm^2^ (95% CI: 1.00, 13.33) to 0.75 cm^2^ (95% CI: 0.23, 3.50) on 30th day of the trial and 0.13 cm^2^ (95% CI: 0.00, 1.08) on day 60th of the trial. According to Wilcoxon signed rank sum test the within-group differences for ulcer area were statistically significant in both groups.

Similar pattern of ulcer size reduction was seen when a cumulative size of the ulcers, which included an ulcer depth, was computed (Table 2). Thus, the average reduction (ARΔ) of ulcer size in RSV-treated patients was 1.96 cm (95% CI: 1.24, 2.53; *P* = 0.000) after 30 days and 3.13 cm (95% CI: 2.10, 4.17; *P* = 0.000) after 60 days of treatment, whereas the corresponding statistics for the placebo group were 0.98 cm (95% CI: 0.55, 1.41; *P* = 0.001) and 2.10 (95% CI: 0.98, 3.21; *P* = 0.002), respectively (Table 2). The mean reduction in the cumulative ulcer size after 60 days using RSV compared with placebo indicated statistically significant treatment difference in multivariable linear regression models (Table 3). Among RSV-treated patients, the complete wound closure took place in 2 out of 14 patients after 30 days and in the 3 other patients after 60 days of intervention. In the placebo group, the complete closure was observed only in 1 out of 10 patients after 60 days of the trial which demonstrates a major difference between placebo and RSV treatment groups. The positive dynamics in the wound healing was also reflected by some reduction in the wound severity assessed by the Wagner scale. Such a reduction either from grade 2 to grade 1 or to complete wound closure took place in 8 out of 14 of the RSV-treated patients versus 3 out of 10 patients in the placebo group.

**Figure 2:**
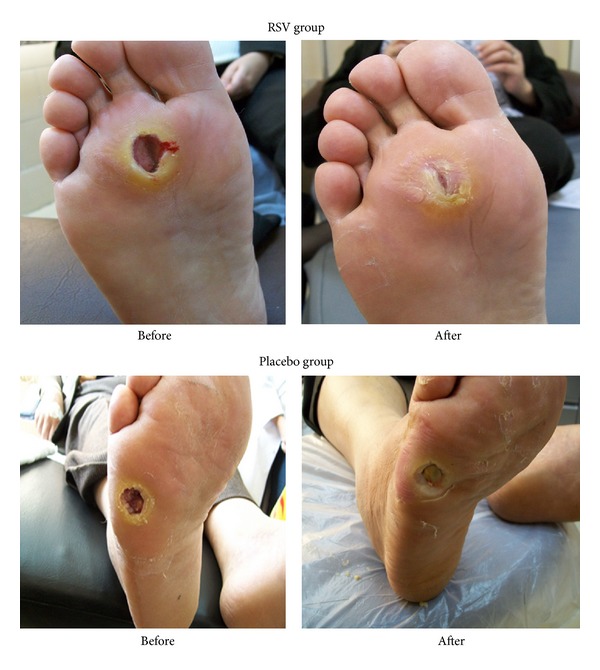
Ulcer images before and after 60-day resveratrol or placebo treatment.

Visual assessment of the ulcers revealed that, besides a higher healing rate and enhanced dynamics of ulcer size reduction, patients in the RSV group tended to have more circularly shaped ulcers with a well-pronounced demarcation zone and healthy pink granulation tissue on the ulcer floor (Figure 2).

### 3.3. Foot Pressure Test

Marginally significant within-group change in plantar pressure of the affected foot was observed in the RSV-treated patients with mean difference (baseline—60 days) of −13.07 kPa (95% CI: −26.78, 0.64; *P* = 0.06). Change in the placebo group was not statistically significant with mean difference of −0.3 kPa (95% CI: −14.95, 14.35; *P* = 0.96), (Table 4). However, a closer look showed that there was some change in the foot plantar pressure for the affected foot in 7/10 patients from the placebo group (versus 10/14 patients in the RSV group). The mean change in the foot plantar pressure after 60 days using RSV in a model adjusted for baseline plantar pressure, affected foot, and ulcer size was 23.04 kPa (95% CI: 2.23, 43.84; *P* = 0.03) indicating statistically significant difference between the groups.

### 3.4. Laboratory Parameters

Among mediators of inflammation which were studied in the patients, only fibrinogen values were affected by RSV treatment. Although some reduction in plasma fibrinogen values was observed in a roughly similar percentage of patients belonging to the placebo (70%) and RSV-treatment (64%) groups, the absolute plasma fibrinogen values declined more noticeably in the RSV-treated patients (from 537.50 ± 45.8 to 418.76 ± 51.3 mg/dL) with an average reduction of 118.9 (95% CI: 12.3, 225.6 mg/dL, *P* = 0.03). In contrast, plasma fibrinogen values in the placebo group changed less significantly—from 568.8 ± 90.3 (before treatment) to 548.10 ± 104.3 mg/dL (after treatment) with average reduction of 20.7 mg/dL (95% CI: 235.5, 276.9, *P* = 0.9), (Table 4). No changes in the CRP levels were found.

There was a clear tendency of reduction in the fasting plasma glucose level in 70% of patients from the placebo and 71% of the RSV group. Mean reduction averaged 19.70 mg/dL (95% CI: −27.12, 66.57; *P* = 0.4) in the placebo group and was more significant in the RSV group [31.57 mg/dL (95% CI: 3.45, 59.69; *P* = 0.03)].

As for the plasma insulin level (Table 4), there were no significant changes among pre- and posttreatment values in neither placebo (*P* = 0.78) nor RSV groups (*P* = 0.3). In compliance with unchanged insulin values, HOMA-IR parameters were not changed in neither placebo (*P* = 0.5588) nor RSV (*P* = 0.1465) groups. However, according to the regression analysis performed (Table 3), insulin levels are important variables for the ulcer size in the RSV group, whereas CRP values are not relevant to study outcome.

Some changes in parameters of lipid homeostasis were found in the patients of both the placebo and RSV groups (Table 4). In particular, 71% of RSV-treated patients had a modest reduction in the concentration of the total cholesterol, from median of 172.50 mg/dL (95% CI: 161.79, 214.96) to median of 166.50 mg/dL (95% CI: 154.91, 192.72, *P* = 0.0166), However, similar decrease took place in 80% of the patients from the placebo group, from median 185.00 mg/dL (95% CI: 163.95, 258.55) to median 153.50 mg/dL (95% CI: 152.22, 204.37; *P* = 0.0273). In both groups, total cholesterol reduction was accompanied by some decrease in the LDL-cholesterol (*P*_pl_ = 0.06; *P*_RSV_ = 0.008) and marginal increase in HDL cholesterol in the placebo group (*P*_pl_ = 0.06; *P*_RSV_ = 0.5).

## 4. Discussion

Here, we show that supplementation of T2DM patients with a proprietary formulation of RSV (*t*-RSV-L, Lycotec Ltd, UK) promotes the reduction of diabetic foot ulcer size when used as an addition to the traditional therapeutic regimen (wound debridement, offloading, establishment of glycemic, and dietary control) applied to newly diagnosed patients with DFS. As can be seen from the placebo group results, establishment of medical supervision and the introduction of a basic therapeutic algorithm in the initial stages of DFS have in themselves an obvious efficiency in the reversal of some clinical and laboratory manifestations of the disease. However, RSV supplementation has an additional positive impact on the progression of DFS, the ulcer size in particular. This effect of RSV emerged in a step-wise manner and had a significant degree of statistical significance when both parameters reflecting the ulcer size (the surface area and cumulative ulcer size) were computed. Some measurable differences in the dynamic of ulcer size changes in the patients representing placebo and RSV groups had already been seen after 30 days of treatment and reached the highest statistical significance at the end of intervention (the 60th day of the trial). It is important to note that the higher values for ulcer size reduction seen in the RSV group resulted in both a higher incidence of full wound closure and reversal of ulcer gradation on the Wagner scale. Moreover, the enhanced dynamics of ulcer size reduction and its closure was accompanied by certain improvements in performance of RSV-treated patients in the foot pressure test. Although the number of patients having value-added performance in the foot pressure test was almost the same in both arms of the study, patients from the RSV group displayed greater changes in values of plantar pressure applied to the affected foot at the end point of the study when compared to the placebo group. These clinical advantages attributable to RSV supplementation were in good agreement with the plasma fibrinogen level reduction which was more pronounced in the RSV-treated patients. Once again, our results emphasize the obvious importance of a regular therapeutic algorithm applied in a timely manner for newly diagnosed patients with DFS. Irrespective of RSV treatment, reductions in ulcer size and plasma fibrinogen level as well as improved parameters of lipid homeostasis and fasting glucose level were seen in the placebo patients. However, RSV supplementation has an obvious additional impact on the study outcomes especially on ulcer size changes and plasma fibrinogen level.

Our study has some significant limitations. First of all, a larger cohort of patients would be beneficial for further evaluation of the effect of RSV on DFS. Secondly, the study was performed on a predominantly poor and medically underserved population of North Africans whose genetic background as well as dietary regimen may have had some effect on the study outcomes. Patients of other ethnicities need to be included in further trials. Thirdly, although the *t*-RSV (*t*-RSV-L) formulation used in our study has been shown to have superior stability under acidic conditions, resistance to oxidation, and enhanced bioavailability when compared to crystallized RSV products (unpublished observation), its further optimization might be very helpful. Finally, our study lacks a considerable body of evidence revealing the mechanism of RSV effect on diabetic ulceration. It can be assumed with some degree of certainty that the effect of RSV on diabetic ulcers arises from various previously described biological effects of the compound on tissue reparation, vascular and neurological functions, inflammatory status, and glucose homeostasis as described by many authors in various in vivo/in vitro systems and reviewed in our previous paper [16]. Indeed, the latest line of evidence suggests that RSV supplementation/treatment has a positive impact on glycemic control in T2DM patients [22], improves inflammatory and fibrinolytic status in patients with a high risk of cardiovascular disease [23], and has a measurable positive effect on endothelial function in patients with metabolic syndrome [24]. On the other hand, it has been reported recently that high-dose RSV supplementation has no appreciable effects on glucose turnover, insulin sensitivity or lipid profile in healthy obese patients [25]. However, any negative projections about metabolic effects of RSV can hardly be made from the results mentioned above since these trials included patients with relatively well-preserved glucose homeostasis to start with. It is obvious that the outcomes of RSV treatment must be closely related to RSV dosage and formulation, duration of treatment, and inclusion criteria for patients enrolled in the studies as well as contaminant pharmacological and/or dietary interventions. Balancing these variables is essential for any attempts at RSV use in the management of T2DM and its complications.

## Figures and Tables

**Table 1:** Baseline characteristics of the enrolled patients.

	Resveratrol group (*n* = 14)	Placebo group (*n* = 10)	*P* value
Age, mean (SD), years	54.0 (10.1)	59.8 (6.6)	0.07^a^
Male, *n*	8	7	0.68^b^
Duration of diabetes, mean (SD), years	15.0 (6.9)	15.2 (9.5)	0.88^a^
Ulcer area, mean (SD), cm^2^	6.9 (8.6)	10.4 (12.9)	0.36^a^
Ulcer length, mean (SD), cm	2.7 (2.6)	2.9 (1.9)	0.44^a^
Ulcer width, mean (SD), cm	1.9 (1.3)	2.7 (1.7)	0.28^a^
Ulcer depth, mean (SD), cm	0.6 (0.2)	0.9 (0.7)	0.33^a^
Ulcer size, mean (SD), cm^†^	5.1 (3.7)	6.4 (3.9)	0.30^a^
Ulcer location, *n*			0.93^b^
Big toe	6	3	
Forefoot	4	4	
Heel	1	1	
Midfoot	3	2	
Ulcer duration, mean (SD), months	18.2 (17.1)	15.0 (11.5)	0.81^a^
Wagner scale, *n*			0.41^b^
Grade 1	9	4	
Grade 2	5	6	
Ulcer type			0.24^b^
Neuroischemic	3	0	
Neuropathic	11	10	
Peak planter pressure, mean (SD), kPa	237.9 (94.6)	176.6 (43.9)	0.07^a^
Hypertension, *n*	8	8	0.39^b^
Retinopathy, *n*	4	2	0.66^b^
Hyperlipidemia, *n*	8	8	0.23^b^
Fasting plasma glucose, mean (SD), mg/dL	201.8 (81.8)	161.2 (72.8)	0.21^a^
Serum insulin, mean (SD), *μ*IU	10.3 (9.5)	15.8 (8.5)	0.04^a^
LDL cholesterol, mean (SD), mg/dL	120.1 (39.3)	139.5 (50.1)	0.31^a^
HDL cholesterol, mean (SD), mg/dL	36.6 (11.5)	34.6 (8.7)	0.70^a^
Fibrinogen, mean (SD), mg/dL	537.5 (171.2)	568.8 (285.6)	0.93^a^
C-reactive protein, mean (SD), mg/L	3.2 (3.4)	2.4 (3.2)	0.46^a^
BMI, mean (SD), kg/m^2^	28.0 (3.5)	29.0 (2.5)	0.50^a^
Smokers, *n*	3	1	0.18^b^
Socioeconomic status, *n*			0.83^b^
Wealthy	1	1	
Middle class	3	3	
Poor	10	6	

^†^Sum of length, width, and depth.

^a^ *P* values were calculated by Wilcoxon-Mann-Whitney test or ^b^Fisher's-exact test.

**Table 2:** Within-group changes in ulcer size by treatment groups (mean differences and 95% confidence intervals).

	Resveratrol group (*n* = 14)	Placebo group (*n* = 10)
Change after 4 weeks		
Ulcer size, cm*	1.96 (1.24, 2.53)	0.98 (0.55, 1.41)
Change in length, cm	0.89 (0.47, 1.32)	0.40 (0.17, 0.63)
Change in width, cm	0.84 (0.50, 1.18)	0.56 (0.30, 0.82)
Change in depth, cm	0.23 (0.11, 0.34)	0.02 (−0.01, 0.05)
Change after 8 weeks		
Ulcer size, cm*	3.13 (2.10, 4.17)	2.10 (0.98, 3.21)
Change in length, cm	1.55 (0.90, 2.20)	0.90 (0.37, 1.43)
Change in width, cm	1.23 (0.85, 1.61)	1.00 (0.44, 1.56)
Change in depth, cm	0.35 (0.22, 0.48)	0.20 (0.02, 0.37)

*Sum of length, width, and depth.

**Table 3:** Multiple linear regression models for ulcer size reduction.

Models	Coefficients (SE)		
	[1]	[2]	[3]
Resveratrol	−1.382* (0.567)	−1.322* (0.562)	−1.338* (0.489)
Baseline ulcer size	0.717*** (0.0917)	0.783*** (0.0691)	0.824** (0.0688)
Baseline insulin		−0.0635 (0.0356)	−0.0506 (0.0319)
Ulcer duration, months		0.0103 (0.0185)	
Baseline Wagner		0.609 (0.660)	
Baseline CRP		−0.247** (0.0864)	−0.222*** (0.0582)
_cons	−0.293 (0.602)	−0.251 (1.157)	0.351 (0.625)

*N*	24	24	24
*R* ^2^	0.835	0.900	0.895
adj. *R* ^2^	0.819	0.865	0.873
rmse	1.348	1.163	1.128

Standard errors in parentheses.

*P < 0.05, **P < 0.01, and ***P < 0.001.

**Table 4:** Within-group changes in selected parameters of diabetic foot ulcer patients over 60 days of the trial. Data are mean differences* (95% CI).

Allocated group at baseline	Resveratrol group (*n* = 14)	Placebo group (*n* = 10)
Peak planter pressure, kPa	−13.07 (−26.78, 0.64)	−0.30 (−14.95, 14.35)
Fasting plasma glucose, mg/dL	31.57 (3.45, 59.69)	19.70 (−27.17, 66.57)
Plasma insulin, *μ*IU	1.29 (−1.48, 4.05)	0.89 (9.46, −5.88)
Plasma fibrinogen, mg/dL	118.93 (12.29, 225.56)	20.70 (−235.48, 276.88)
CRP, g/L^†^	−0.25 (−1.50, 1.50)	−0.05 (−2.50, 1.40)
Total cholesterol, mg/dL	17.29 (3.52, 31.05)	32.60 (4.29, 60.91)
HDL cholesterol, mg/dL	−1.43 (−6.27, 3.42)	3.40 (−0.11, 6.91)
LDL cholesterol, mg/dL	20.0 (6.2, 33.8)	23.80 (−1.36, 48.96)

*Baseline-60 days.

^†^Median difference with robust CIs.
